# Single-Crown, Short and Ultra-Short Implants, in Association with Simultaneous Internal Sinus Lift in the Atrophic Posterior Maxilla: A Three-Year Retrospective Study

**DOI:** 10.3390/ma13092208

**Published:** 2020-05-11

**Authors:** Giorgio Lombardo, Mauro Marincola, Annarita Signoriello, Giovanni Corrocher, Pier Francesco Nocini

**Affiliations:** 1 School of Dentistry, Department of Surgery, Dentistry, Paediatrics and Gynaecology (DIPSCOMI), University of Verona, Piazzale L.A. Scuro 10, 37134 Verona, Italy; giorgio.lombardo@univr.it (G.L.); giovanni.corrocher@univr.it (G.C.); pierfrancesco.nocini@univr.it (P.F.N.); 2 Research Department, Dental Implant Unit, Faculty of Dentistry, University of Cartagena, Cartagena 130001, Colombia; mauromarincola@unicartagena.edu.co

**Keywords:** bone gain, crestal bone height, implant survival, internal sinus lift, maxilla, short implant, single crown, ultra-short implant

## Abstract

As the atrophic posterior maxilla often presents serious limitations for dental implant procedures, a minimally invasive technique was proposed. The study aimed to retrospectively evaluate the outcomes of short and ultra-short locking-taper implants, placed in combination with a modified osteotome sinus floor elevation procedure (internal sinus lift technique) in the posterior maxilla. A total of 31 patients received 51 locking-taper implants. Clinical and radiographic examinations were performed before treatment, at loading time, and after three years. Seven implants of 8.0 mm, 23 implants of 6.0 mm, and 21 implants 5.0 mm in length were rehabilitated with single-crown restorations. Implant survival at three-year follow-up was 96.08%. Pre-operative residual crestal bone height of 5.2 (1.41) (median (interquartile range)) mm increased to 7.59 (1.97) mm at the 36-month follow-up, with an average intra-sinus bone height gain of 3.17 ± 1.13 (mean ± standard deviation) mm. Mean peri-implant crestal bone loss was 0.29 (0.46) mm and mean first bone-to-implant contact point shifted apically to 0.12 (0.34) mm. It can be suggested with confidence that implants used in the study, placed in conjunction with an internal sinus floor elevation technique, can be restored with single crowns as a predictable treatment for the edentulous regions of the posterior maxilla.

## 1. Introduction

Reduced bone volumes characterized by severe atrophy and increased sinus pneumatization are often encountered in the posterior maxilla, especially in sites involved in extraction of periodontally compromised teeth [1]. A radiological study [2] reported that edentulous first and second molar sites showed a mean residual crestal bone height (RCBH) of 3.3 ± 2.2 mm and 4.5 ± 2.4 mm, respectively; furthermore, the prevalence of sites with RCBH less than 5 mm in these areas was 73.1% and 54.2%, respectively [2].

As bone in the posterior maxilla has poor quality and large marrow spaces, its anatomical and physiological features often represent serious restrictions. To overcome these limitations, a number of procedures, such as lateral or transcrestal maxillary sinus floor elevation [3] and bone substitutes (autografts, allografts, xenografts, and synthetic biomaterials) [4], were proposed for sinus grafting and for augmenting bone volumes in the posterior maxilla, to enable dental implant placement [5,6,7,8]. Though these procedures often presented a high rate of implant survival and bone levels’ stability over time [9], they were not always well accepted by the patients because of expense, increased post-operative morbidity, high risk of infection (fistula with pus or abscess, frequently caused by infection of the grafted material), and prolonged healing time [10,11,12].

In 1994, a less invasive procedure called osteotome sinus floor elevation (OSFE) was proposed by Summers [13,14,15,16]. Although this procedure was historically recommended and was proved to be effective for patients with at least 7 mm of bone below the sinus floor, recent studies have established comparable implant survival in the case of sites with an even lower RCBH [17,18,19]. Nevertheless, when the limit of 5.0 mm of RCBH was exceeded, implant survival seemed to significantly drop [20,21,22,23] from 96% to 85.7% [20]. Furthermore, a meta-regression analysis [24] postulated that standard implants (longer than 8.0 mm), placed simultaneously with the OSFE procedure in implant sites with RCBH less than 5.0 mm beneath the sinus, seemed to be at a greater risk for failure.

Following the development of innovative implant design and surface textures, literature reviews and meta-analysis of the last decades [25,26,27] have supported the use of implants 6.0 mm and 8.0 mm in length (defined as short implants) in the treatment of resorption in the posterior maxilla. These recent studies [25,26,27] have also reported high implant survival for these implants, placed via a crestal approach, and using the OSFE technique in patients with even less than 5.0 mm of RCBH. Though these investigations have considered short implants supporting different types of restorations, the evidence concerning clinical outcomes of short implants, placed in conjunction with an OSFE technique and supporting nonsplinted single crowns in the atrophic posterior maxilla, remains scarce. It is, however, suggested that the use of single-crown restorations confers additional potential benefits, such as better oral hygiene access, improved acceptance, and patient comfort [28].

With due consideration for the relatively short-term follow-up, the study aimed to retrospectively evaluate the outcomes of locking-taper implants, 8.0 mm, 6.0 mm, and 5.0 mm in length, placed in combination with a modified osteotome sinus floor elevation procedure, called internal sinus lift technique (ISL). We hypothesized that these implants, restored with single crowns, can represent a successful therapy in cases of extremely reduced RCBH.

## 2. Materials and Methods

### 2.1. Study Design and Inclusion Criteria

The patients were recruited and treated, between January 2014 and January 2015, with implant-supported single crowns for edentulism (tooth loss caused by trauma, caries, or periodontal disease) in the posterior maxilla at the Dental and Maxillo-Facial Surgery Clinic at the University of Verona (Italy). A retrospective study with a 36-month follow-up [29,30] was conducted between June and September 2018. The University Institutional Review Board approved the retrospective study (Protocol “SINUSLIFT”, 23/05/18). The nature and aim of the study, together with the anonymity in the scientific use of data, were clearly explained in a written, informative consent form, which was signed by every patient. All clinical procedures were performed in accordance with the Declaration of Helsinki and the good clinical practice guidelines for research on human beings.

To be included in the study, patients had to have at least one 5.0 mm, 6.0 mm, or 8.0 mm in length locking-taper implant [29,30], which had been placed in a partially edentulous posterior maxilla in combination with an ISL procedure and which supported a single crown. In addition, the RCBH must have been equal to or less than 6.0 mm and a crestal bone thickness must have been of at least 6 mm, as determined by CBCT (cone beam computed tomography) scan measurements.

Exclusion criteria considered were: The presence of active infection at an implant site; ASA status III (according to the American Society of Anesthesiologists’ classification [31]), that is severe systemic diseases or substantive functional limitations which contraindicated implant surgery (such as drug or alcohol abuse, uncontrolled diabetes mellitus, immunosuppression or immunodepression, severe autoimmune diseases, treatment or past treatment with intravenous amino-bisphosphonates for metastatic bone diseases, radiotherapy to head or neck within two years prior to treatment, history of malignancy or chemotherapy within the previous year, treatment with oral amino-bisphosphonates for more than three years, morbid obesity, active hepatitis, severe renal disease, severe cardiovascular conditions, recent history of myocardial infarction (MI) or transient ischemic attack (TIA)); ASA status IV, V, and VI; history of sinus surgery; acute or chronic maxillary sinusitis; oro-antral fistulae; untreated periodontitis; poor oral hygiene and motivation; current pregnancy or lactation; heavy smoking (more than 25 cigarettes per day) [32]; and severe clenching or bruxism.

### 2.2. Surgical Protocol

All treatments and visits were carried out by two experienced periodontal surgeons.

Pre-operative assessment consisted of clinical and radiographic evaluation. Panoramic radiographs were used for initial screening, followed by CBCT scans to precisely quantify the amount of available bone under the maxillary sinus. Furthermore, an intraoral radiograph performed with parallel technique was made to determine the baseline RCBH and to allow future comparison with the CBCT scan measurement. When the operative site involved more than one tooth, diagnostic casts for the creation of a mucosal supported surgical guide were made. One month before surgery each patient underwent a full-mouth session of scaling and root planing, using mechanical and hand instrumentation, and received personalized oral hygiene instructions [33].

A pre-operative medication consisting of 2 g of Augmentin (875 mg amoxicillin plus 125 mg clavulanic acid), or 1 g of Klacid (Clarithromycin 500 mg) if allergic to penicillin, was given one hour before surgery [34]. All surgical procedures were performed under local anaesthesia, using only Articain 4% with adrenaline 1:100,000 (Citocartin) or Articain 4% with adrenaline 1:100,000 (Citocartin) associated with oral sedation (Halcion 0.25 mg).

After a midcrestal incision, buccal and palatal full-thickness flaps were reflected. Vertical releasing incisions were made only if necessary. The recipient sites were marked with a 2.0-mm-round drill. If the edentulous space involved more than one tooth, the mark was made in accordance with the pre-prepared surgical templates. The osteotomy was initiated using a 2.0-mm-diameter pilot drill to a depth of 0.5 to 1.0 mm from the sinus floor, while being guided by pre-operative radiographs and the CBCT. The expansion of the osteotomy sites continued with successively larger dedicated manual reamers to create an osteotomy of 5.0 mm diameter and 1.0 mm from the sinus floor. The sinus floor fracture was obtained by inserting a 5.0-mm sinus lift osteotome into the osteotomy to the level of the sinus floor and gently tapping the osteotome with a mallet to create a hairline fracture in the floor of the sinus. Great attention was given to avoid perforation of the sinus membrane. After completion of this procedure, the integrity of the Schneiderian membrane was manually confirmed by gentle sounding with a blunt tipped depth gauge. The membrane was then elevated by placing a synthetic bone graft material into a syringe and injecting it into the osteotomy. As the column of graft material was advanced in the osteotomy, it gently lifted the sinus membrane to the desired height. Implants were placed immediately after the sinus elevation using an implant inserter and using the implant to further raise the sinus floor.

Short implants (8.0 and 6.0 mm in length) or ultra-short implants (5.0 mm in length) were utilized in this study. The locking-taper (Morse taper or Morse cone) dental implant system (Bicon Dental Implants, Boston, MA, USA, designed in 1985) has an implant-abutment interface (IAI) connection, which is impervious to bacterial penetration or infiltration [35]. The implant system also includes a convergent crest module, platform switching, plateau root-form design, and an Integra CP^TM^ surface (Hydroxylapatite treated and acid etched).

**Figure 1:**
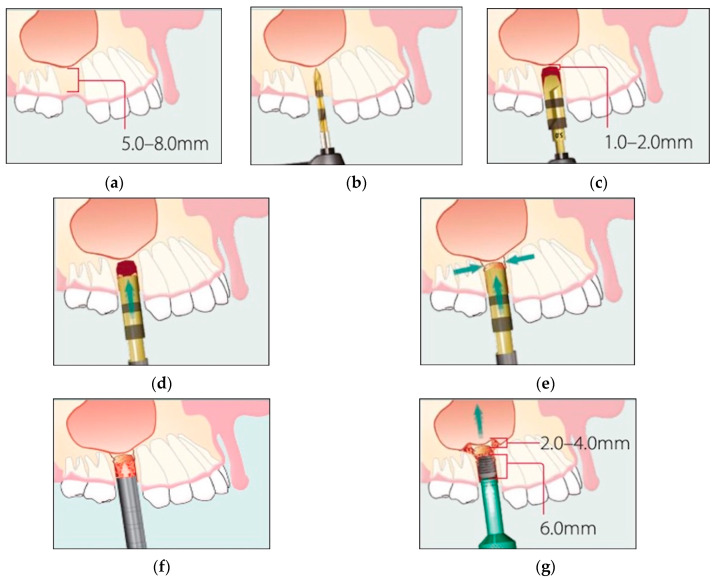
(**a**–**g**). Schematic drawing of internal sinus lift (ISL) technique with Bicon dental implant. (**a**) Note the minimal residual bone depth of 5.0-8.0 mm. (**b**) Prepare osteotomy beginning with the 2.0-mm pilot drill. (**c**) Continue to prepare osteotomy with successively larger reamers to the extent that 1.0-2.0 mm of undisturbed bone remains below the sinus floor (a 5.0-mm-diameter implant has been chosen for this case). (**d**) Place a 5.0-mm sinus lift osteotome into the osteotomy and engage the area slightly below the sinus floor. (**e**) Gently tap the osteotome and create a hairline fracture around the floor of the osteotomy. (**f**) Place a bone graft material into the socket. (**g**) Introduce the implant into the osteotomy site with the implant inserter and use the implant to raise the sinus floor.

Before implant placement, a sinus lift temporary abutment was inserted into the implant to prevent the implant from migrating into the sinus. The flaps were accurately sutured, allowing for a primary wound closure, and all implants were left submerged during the following six-month healing period. Immediately after flap closure, periapical radiographs, which would serve as baseline for future comparison, were made with the paralleling technique [29,30]. Figure 1a–g reports a schematic drawing of the implant procedure.

Patients received detailed post-operative instructions, along with antibiotic and analgesic prescriptions. After one week, patients were monitored for evidence of post-operative swelling and/or headaches. The sutures were removed after two weeks, and patients were instructed not to use removable dentures during the six-month healing period.

### 2.3. Prosthetic Protocol and Follow-Up Evaluation

After six months, implants were surgically uncovered, healing abutments were placed, and the mucosal flaps re-adapted and sutured around the healing abutments. After three weeks of soft tissue healing, definitive impressions were taken, using a polyether material. Definitive single-crown porcelain or composite restorations were delivered within two weeks. The technique used was the IAC (Integrated Abutment Crown), in which the abutment and the crown material are extra-orally, chemo-mechanically bonded; therefore, there was no need for cement, and the implant and IAC are connected with a screwless locking-taper connection [36].

**Figure 2:**
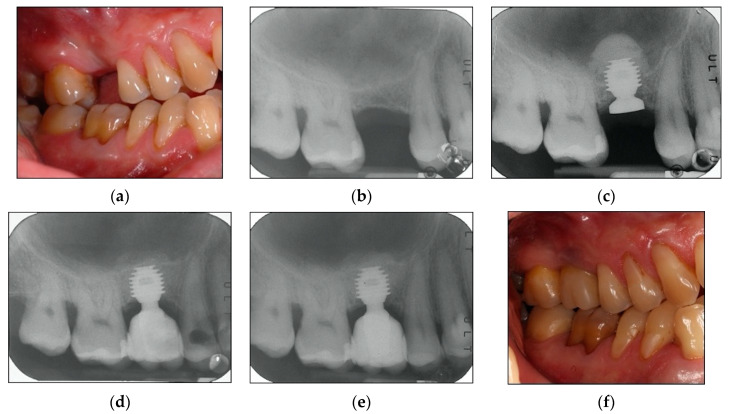
(**a**–**f**). Clinical case: Single implant placed in 1.6 site. (**a**) Clinical photograph before implant placement, missing first molar. (**b**) Pre-operative radiograph before implant placement in site 1.6. See minimal bone in implant site. (**c**) Radiograph obtained at implant placement. See short implant with sinus lift temporary abutment designed to prevent displacement of the implant into the sinus, also see augmented sinus floor. (**d**) Radiograph obtained at time of loading. See augmented sinus floor, also see the radiolucent area in the crown of the second bicuspid, which was a lost restoration. (**e**) Radiograph obtained at three-year follow-up. See stable bone levels, also see the replaced restoration in the crown of the second bicuspid. (**f**) Clinical photograph at three-year follow-up. See stable clinical conditions.

**Figure 3:**
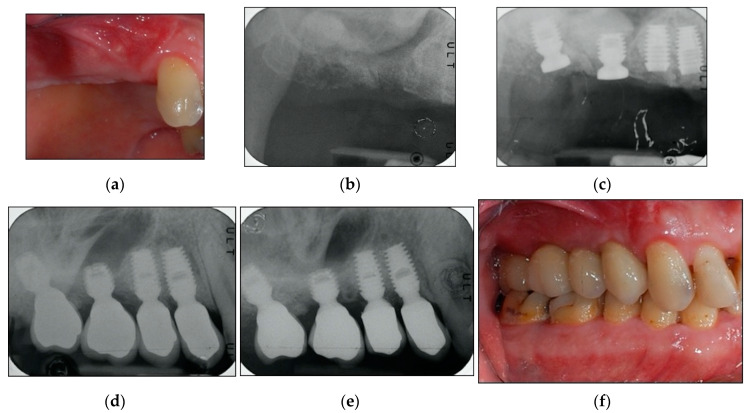
(**a**–**f**). Clinical case: Four implants placed in 1.4, 1.5, 1.6, and 1.7 sites. (**a**) Clinical photograph before implants placement. (**b**) Pre-operative radiograph before implants placement in sites 1.4, 1.5, 1.6, and 1.7. See minimal bone levels. (**c**) Radiograph obtained at time of implants placement. Two of the implants have sinus lift abutments. Augmented sinus visible. (**d**) Implants restored. Radiograph obtained at time of loading. (**e**) Radiograph obtained at the three-year follow-up. See stable bone levels both for sites with augmented sinus floor and for sites without it. (**f**) Clinical photograph at three-year follow-up. See stable clinical conditions.

**Figure 4:**
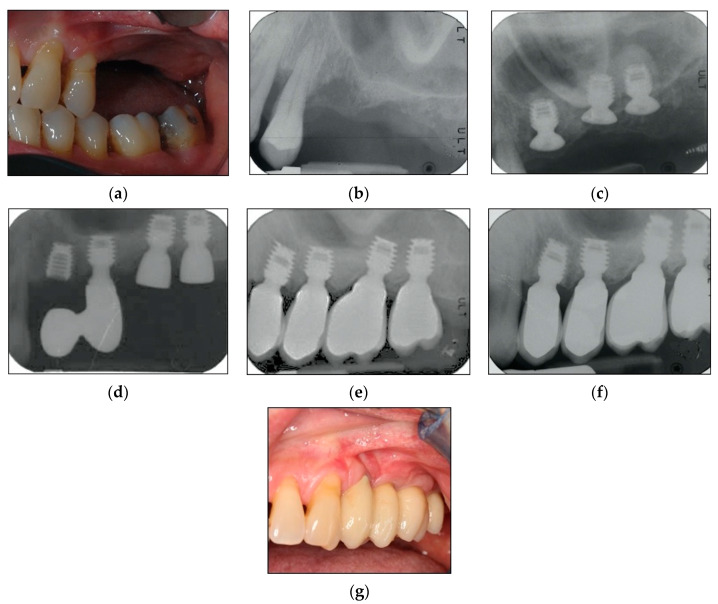
(**a**–**g**). Clinical case: Four implants placed in 2.4, 2.5, 2.6, and 2.7 sites. (**a**) Clinical photograph before implants placement. (**b**) Pre-operative radiograph before implants placement in sites 2.5, 2.6, and 2.7. (**c**) Radiograph obtained at implants placement. See augmented sinus floor. (**d**) Radiograph obtained at loading time. Another implant was placed in site 2.4 (the extracted tooth had grade 3 mobility and was extremely compromised). Site 2.4 was temporarily restored using the implant in 2.5 site to support a cantilever prosthesis. (**e**) Radiograph obtained at loading time. See definitive restorations for all implants. (**f**) Radiograph obtained at three-year follow-up. See stable bone levels. (**g**) Clinical photograph at three-year follow-up. See stable clinical conditions.

A maintenance program was designed to provide patients a professional oral hygiene session every four months [33] and home care procedures were reinforced. At each recall appointment, occlusion was assessed and adjusted as necessary; prosthetic restorations were checked for loosening, chipping, or other types of complications. Clinical assessment of peri-implant soft tissues and radiographic examinations were performed after three years of follow-up from loading time [29,30]. By way of illustration, Figure 2, Figure 3 and Figure 4 report some radiographic cases.

### 2.4. Study Variables and Outcomes

Implant lengths considered in this study were 8.0 mm, 6.0 mm, and 5.0 mm; implant diameters were 4.0 mm, 4.5 mm, 5.0 mm, and 6.0 mm. Covariates included were: Sex, age, smoking history, history of periodontal disease, ASA status, number of oral hygiene sessions per year, interproximal access for oral hygiene, tooth site, prosthetic material, and crown-to-implant ratio (CIR) [29,30].

The main outcome was implant survival after three years of follow-up. Implant failure was considered as the need for implant removal either before loading (due to no osseointegration) or after loading (due to excessive bone loss). Implant survival was considered as the implant’s state of being in function at the three-year follow-up evaluation, that is, symptom-free, without mobility, radiolucency, or bone loss so severe as to warrant implant removal [29,37,38,39].

A secondary outcome included variations of peri-implant bone levels and sinus floor level, which were measured through digitally scanned intraoral radiographs, performed with parallel technique [40], using Rinn centering devices (Rinn XCP Posterior Aiming Ring-Yellow, Dentsply, Elgin, IL, USA), immediately after implant placement, at healing abutment placement, at prosthetic loading, and after three years of loading. The implant-abutment interface (IAI) was taken as a reference for measurements [29].

A descriptive analysis of crestal bone level (CBL, average bone level around implants at mesial and distal sides, in mm) and first bone-to-implant contact (F-BIC, in mm) [41,42,43], along with their variations ∆CBL (average bone loss) and ∆F-BIC (average apical shift of the first bone-to-implant contact point position) was conducted. These values were determined based on changes that took place between loading time and the three-year follow-up time, according to covariates. CBL was measured on mesial and distal sides as the linear distance between the IAI and the highest point of the interproximal bone crest parallel to the lateral sides of the implant body. A positive value was given when the crest was located coronally to the IAI and a negative value was given when the crest was located apically to the IAI. F-BIC was defined as the first most coronal bone-to-implant relationship visible at the first line of contact, on both mesial and distal sides. If F-BIC matched with IAI, the measurement was 0. If it was located apically, the measurement was a positive value. For every implant, an average (av) mesial-distal value (av-CBL and av-FBIC) was calculated at each examination interval [29].

Furthermore, as described in the literature [44], implants were divided into two groups on the basis of presenting a crown-to-implant ratio (CIR) less than or greater than 2. The crown height was measured on the radiograph immediately after the prosthetic loading, from the most occlusal point to the IAI. Anatomical CIR [44] (in which the fulcrum is positioned at the interface between the implant shoulder and the crown-abutment complex) was calculated by dividing the digital length of the crown by the digital length of the implant [29].

Sinus floor level (SFL) was measured on the mesial, central, and distal point of each implant, as the linear distance between the IAI and the sinus floor. For each implant, at each examination interval, an average (av) mesial-distal-central value for sinus floor level (av-SFL) was calculated. The sum of av-CBL and av-SFL was calculated as the residual crestal bone height (RCBH). The vertical increase in height of the implant site (intra-sinus bone height gain, IBHG) was also calculated as the difference of the RCBH with the pre-operative RCBH, in order to obtain the final crest height [45,46].

Measurements were assessed with the aid of a software program (Rasband, W.S., ImageJ, U.S. National Institutes of Health, Bethesda, MD, USA) which uses a measuring tool in conjunction with a magnification tool. To correct the distortion of the radiographic image, the apparent size of each implant (measured directly on the radiograph) was compared with the actual length of the implant, to determine with adequate precision the amount of change in the crestal bone around each implant. The measurements were made to the nearest 0.01 mm [29]. Beyond that, the results from the pre-operative periapical radiographs were compared with those of pre-operative CBCT scans. If disagreements were present between the values, the CBCT values were chosen and served as reference for future comparison with the radiographs. One dentist, who was not involved in the treatment of the patients, completed all the measurements on periapical radiographs and CBCT scans; the observation intervals of the radiographs were masked to the examiner. Before the start of the study, this investigator was calibrated for intra-examiner adequate levels of accuracy and reproducibility in recording the radiographic parameters. Three radiographs were used for this purpose: Duplicate measurements for CBL, F-BIC, SFL, and CIR were collected with an interval of 24 h between the first and second recording. The intra-class correlation coefficients, used as a measure of intra-examiner reproducibility, had to be greater than 0.8 [29].

**Figure 5:**
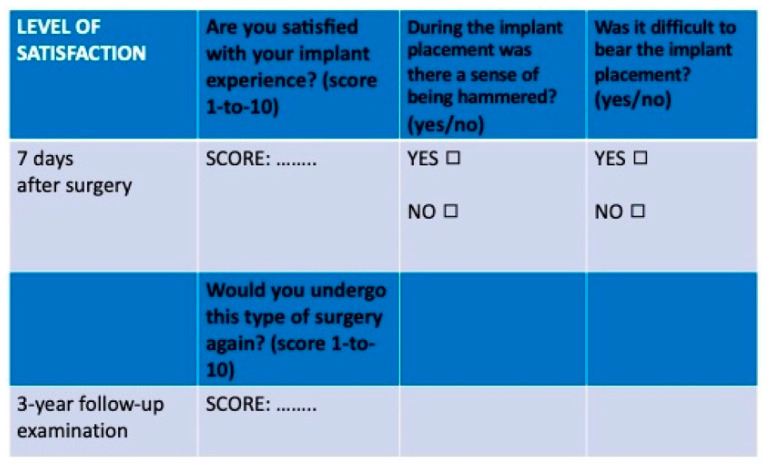
Level of satisfaction: Each patient had to report it seven days after surgery and at three-year follow-up examination.

After seven days and at the three-year follow-up examination, each patient was asked to quantify the level of their satisfaction (Figure 5), on a 1-to-10 point visual analogue scale (VAS) [47], with the implant experience and considering the potential benefits, if they would undergo this type of surgery again.

### 2.5. Statistical Analysis

For data collection, a database including all patients evaluated in the study was created with Microsoft Excel. All data analysis was carried out using Stata v.13.0 for Macintosh (StataCorp., College Station, TX, USA) [29,48]. The normality assumptions for continuous data were assessed by using the Shapiro-Wilk test; mean and standard deviation (SD) were reported for normally distributed data [mean ± SD], median, and interquartile range (iqr) otherwise (median(iqr)). For categorical data, absolute frequencies, percentages, and 95% confidence intervals were reported. The association between categorical variables was tested with χ2 test; if any of the expected values was less than 5, a Fisher’s exact test was performed. The comparison between the means of continuous variables in two different times was performed by using paired Student’s “t” test or Wilcoxon matched-pairs signed-rank test. The comparison between the means of two different groups was performed using unpaired Student’s “t”, or Wilcoxon rank-sum test. The comparison of the means among more than two groups was done using one-way analysis of variance (ANOVA) or Kruskal–Wallis equality-of-populations rank test as appropriate. Bonferroni correction for multiple comparison was applied. Significance level was set at 0.05.

## 3. Results

### 3.1. Demographics

Thirty-one patients (20 women and 11 men) were included for the retrospective study according to inclusion and exclusion criteria. Mean age at placement was 53.59 ± 10.48 years (range 34–75). Twenty patients (with 37 implants) had lost their teeth due to periodontal disease (in some cases this was self-reported, in others it was determined through patient’s dental records), while 11 patients (with 14 implants) had lost their teeth for other reasons.

Of the implants, 41.18% were 5.0 mm, 45.1% were 6.0 mm, and 13.73% were 8.0 mm in length. Most of the implants (74.51%) were placed in the molar area. Implant diameters were 4.0 mm (3.92%), 4.5 mm (29.41%), 5.0 mm (56.86%), and 6.0 mm (9.8%), respectively. Of the implants, 39.22% and 60.78% were placed in patients, respectively, with ASA status I and II. All implants were restored with single crowns, 44 of them with porcelain crowns, and seven with resin crowns. Mean CIR was 1.99 ± 0.44 (range 1.07–2.85). A CIR ≥ 2 prevalence was estimated in 49.02% of the implants. Significant differences (*p* = 0.01) for CIR among length groups were found: 1.3 ± 0.15 (range 1.20–1.61), 1.92 ± 0.25 (range 1.42–2.32), and 2.27 ± 0.44 (range 1.07–2.85) for implants 8.0 mm, 6.0 mm, and 5.0 mm in length.

The implant distribution was analyzed according to length definition (8.0, 6.0, and 5.0 mm). The overall descriptive statistics for the study variables are presented in Table 1.

### 3.2. Implant Survival

At the uncovering stage, all the implants were osteo-integrated and no early failures were detected. Two implants were lost after functional loading (late failures due to excessive bone loss) in two patients (one with history of periodontitis) at the three-year follow-up. Failures occurred in 4.5 × 6.0 mm and 5.0 × 6.0 mm implants, respectively, with a CIR of 1.43 and 2.17. The overall implant survival 36 months after loading was 96.08%. There were no statistically significant differences (*p* = 0.62) between length groups (100%, 91.3%, and 100% for implants 8.0 mm, 6.0 mm, and 5.0 mm in length, respectively). No association was found between survival and failure groups, nor in any of the considered covariates, as reported in Table 2.

### 3.3. Radiographic Bone Levels

Average crestal bone levels were stable between loading time and follow-up time, with a mean ∆CBL of 0.29 (0.46) mm and a mean ∆F-BIC of 0.12 (0.34) mm. Outcomes regarding CBL, F-BIC, RCBH, and IBHG at each time interval are listed in Table 3.

Even if statistically significant differences between time intervals were found, we can assume these variations as not clinically relevant: Average values obtained for CBL, F-BIC, RCBH, and IBHG after three years of follow-up are compatible with bone levels’ stability. As implant length was considered a clinically relevant covariate, the comparison for CBL, F-BIC, RCBH, and IBHG between length groups is reported in Table 4: No statistically significant differences among length groups were found for any of the variables at any time interval.

### 3.4. Patients’ Level of Satisfaction

Seven days following the surgery, when questioned about their level of satisfaction with the implant procedures, 14 patients (45.16%) gave a score between 9 and 10, 15 (48.39%) gave a score between 6 and 8, and two (6.45%) gave a score of 5; furthermore two patients referred to “being hammered” and two reported “difficult in bearing”. At three-year recall appointment, when asked the same question and whether they would undergo the surgery again, none of them retained a negative memory of the entire procedure. On the contrary, they all said that they would undergo the treatment again, and the average score was higher compared to the average score related to seven days after surgery (Table 5). More precisely, 25 patients (80.65%) gave finally a score between 9 and 10 and six (19.35%) gave a score between 7 and 8. Statistically significant differences (*p* < 0.001) were found between the first and second time of evaluation (Table 5).

## 4. Discussion

According to a traditional point of view, a residual bone height of at least 5.0 mm in the atrophic posterior maxilla is considered a predictable option for implant placement when done in tandem with a sinus augmentation using the OSFE technique [17,18,19]. A few studies in the literature claimed predictability for standard implants’ (longer than 8.0 mm) placement in conjunction with an immediate OSFE procedure in RCBH even less than 4.0 mm [49,50]. Nevertheless, the majority of the authors reported lower survival in cases of implant placement in RCBH less than 5.0 mm, strongly suggesting delayed implant placement in such cases, to allow proper graft healing and to achieve primary implant stability [20,51,52,53,54].

In addition, to facilitate the placement of implants in minimal volumes of crestal bone, several recent studies have shown that short implants (8.0 and 6.0 mm in length) with rough surface are capable, within certain limits, of tolerating high CIR (shown below), providing stable bone levels as well as implant survival comparable to those of standard implants in the long term [41,42,43,55,56].

It was also demonstrated that the Schneiderian membrane can properly support elevation of 4.0 mm without perforation [57]. On this basis, the use of short implants, requiring only a minimal amount of sinus floor elevation, while reducing the risk of sinus perforation [58], might represent the most prudent and conservative surgical approach to rehabilitate the perennially problematic atrophic posterior maxilla. The use of short implants in conjunction with the OSFE technique in the treatment of RCBH equal or inferior to 5.0 mm was proposed with favorable results [45]. It is noted, however, that there is still a lack of data regarding the long-term efficacy of this therapeutic solution.

In this study, favorable outcomes for implant survival and bone levels’ stability were found at three-year follow-up, despite the prevalence of CIR > 2 in almost half of the sample size and the presence of patients with history of periodontitis accounting for a high percentage of implants. These findings are consistent with those of two recently published studies, where short implants had been placed in a similar anatomical setting. A randomized controlled trial (RCT) by Si et al. [45] followed 41 short implants placed in association with OSFE in 41 patients who had an initial average RCBH of 4.63 mm. After three years, an implant survival of 95.2% and 95% and an IBHG of 3.17 mm and 3.07 mm were reported for a group with and another group without grafting, respectively. A study by Teng et al. [46], compared to the present investigation, presented slight differences; specifically, the crestal sinus elevation methodology used trephine burs instead of osteotomes. In that study, 50 short implants were placed in extremely reabsorbed maxilla and reported a survival rate of 100%, with an IBHG of 4.4 mm and a final RCBH of 7.77 mm after a short-term follow-up.

It was recently demonstrated [59] that the type of implant-abutment connection and the implant body design may play an important role in peri-implant soft tissue health and hard tissue stability. The locking-taper connection and the plateau-body design of the implants utilized in the present study seem to assure a functional sealing of the implant-abutment connection [35] and create a favorable influence on bone level stability, even in the presence of high CIR [29,30]. Consequently, the implant design results in proper distribution of augmented loadings and lateral forces, which, in turn, preserves the limited amount of crestal bone available in the atrophic posterior maxilla, despite the presence of single-crown restorations characterized by increased CIR [42]. Therefore, multiple splinted crowns may no longer be considered as an essential therapeutic choice in presence of short and ultra-short implants with a similar design [42]. Nevertheless, more studies with a longer follow-up and larger samples are necessary to validate these approaches. In the study at hand, the 5.0-mm implants presented a significantly higher mean CIR compared to the other length groups and no failures after three years. This might suggest that not only short but also ultra-short implants are able to support large single crowns and can be considered a rational treatment for the atrophic posterior maxilla. That said, these findings must take into account that studies regarding the use of 5.0-mm-length implants to support single crowns are currently scarce and heterogeneous [29,30,38,60].

It also must be stressed that all patients in the study underwent regular supportive care in the form of full-mouth ultrasonic debridement every four months throughout the study period and that proper periodontal treatment prior to implant placement was the standard of care, necessary for patients with active periodontal disease: For this group of patients, a strict maintenance program is even more strongly recommended for the long-term success of short implants [33].

Another point related to patients’ management is the premedication with antibiotics before and after implant placement to prevent infections. Even if the routine use of pre- and post-operative antibiotics in oral surgery remains controversial [34], we assumed that the surgical procedures adopted in this study presented high risk of infection. Thus, antibiotic prophylaxis was required [61].

The main complication related to the OSFE procedure described in literature [62] is the repeated hammering of the osteotome, which is intended to compact the column of bone substitute material while using it to create a controlled fracture of the sinus floor. This process causes a localized apical dislocation of the sinus floor while maintaining the integrity of the Schneiderian membrane. Furthermore, tapping with a hand mallet may induce BPPV (benign paroxysmal positional vertigo): This may be surprising and somewhat unpleasant for patients who have never experienced this form of vertigo [62,63,64]. In our study, even though some patients referred to “being hammered” or having “difficulty in bearing”, when asked about their experience seven days after surgery, none of them actually reported experiencing vertigo. At the three-year recall appointment, none of them had retained a negative memory of the entire procedure: All said that they would readily undergo the same procedure in order to obtain the same benefits.

Finally, our study does present some critical issues relative to its retrospective nature: The small sample size, a three-year follow-up evaluation, a non-homogeneous distribution among implant length groups, and a single center (the University Dental Clinic). On the other hand, a one-year interval (January 2014–January 2015) for patient recruitment could be a favorable point in excluding any significant variations of technique. Most of the patients enrolled in the study were characterized by a history of periodontal disease; this was potentially a critical limitation for the study, but it was not a significant issue for implant survival.

Despite the limitations discussed above, the main strength of our study comprises a positive assessment: Three years after loading, a high proportion of locking-taper implants (96.08%) placed in conjunction with an internal sinus floor elevation technique, restored with single crowns and having a moderately disproportionate CIR, survived with stable crestal bone levels. However, further long-term investigations (five-year follow-up or longer) with a prospective approach, a major homogeneity in length-group distribution, and a larger sample size are needed to corroborate our results on ultra-short implants in the posterior atrophic maxilla.

## 5. Conclusions

Within the limits of the present retrospective short-term study, our clinical and radiographic outcomes suggested that short and ultra-short locking-taper implants, placed in conjunction with an ISL technique and restored with single crowns, can be considered a predictable treatment for edentulous posterior maxillary regions with RCBH less than 6.0 mm.

## Figures and Tables

**Table 1:** Overall characteristics of implants placed: Length-group distribution according to study variables. Age at follow-up, months at loading/follow-up time, and oral professional hygiene/year are presented as [mean ± SD]; for all other variables, values are presented as n (%).

VARIABLE	Overall (N = 51)		5 mm (N = 21)		6 mm (N = 23)		8 mm (N = 7)		*p*-Value
	n	%	n	%	n	%	n	%	
**SEX**									
Male	20	39.22	9	42.86	7	30.43	4	57.14	0.42
Female	31	60.78	12	57.14	16	69.57	3	42.86	
**AGE AT FOLLOW-UP**	56.22 ± 18.86		56.68 ± 11.11		58.04 ± 9.99		62.70 ± 9.26		0.35
**MONTHS AT LOADING TIME**	11.50 ± 5.54		12.47 ± 5.49		11.30 ± 5.70		9.28 ± 5.18		0.41
**MONTHS AT FOLLOW-UP TIME**	42.86 ± 20.33		41.04 ± 17.52		43.73 ± 23.31		45.42 ± 20.14		0.85
**SMOKING**									
No	42	82.35	15	71.43	20	86.96	7	100.00	0.23
Yes	9	17.65	6	28.57	3	13.04	0	0.00	
**ASA STATUS**									
I	20	39.22	7	33.33	12	52.17	1	14.29	0.17
II	31	60.78	14	66.67	11	47.83	6	85.71	
**ORAL HYGIENE SESSIONS/YEAR**	3.11 ± 1.05		2.95 ± 1.16		3.30 ± 0.97		3.00 ± 1.00		0.52
**INTERPROXIMAL ORAL HYGIENE**									
No	10	19.61	3	14.29	5	21.74	2	28.57	0.74
Yes	41	80.39	18	85.71	18	78.26	5	71.43	
**HISTORY OF PERIODONTAL DISEASE**									
No	14	27.45	5	23.81	8	34.78	1	14.29	0.57
Yes	37	72.55	16	76.19	15	65.22	6	85.71	
**TYPE OF TOOTH**									
Premolar	13	25.49	5	23.81	3	13.04	5	71.43	0.01
Molar	38	75.51	16	76.19	20	86.96	2	28.57	
**IMPLANT DIAMETER**									
4 mm	2	3.92	2	9.52	0	0.00	0	0.00	<0.001
4.5 mm	15	29.41	0	0.00	8	34.78	7	100.00	
5 mm	29	56.86	14	66.67	15	65.22	0	0.00	
6 mm	5	9.8	5	23.81	0	0.00	0	0.00	
**PROSTHETIC MATERIAL**									
Resin	7	13.73	5	23.81	1	4.35	1	14.29	0.15
Porcelain	44	86.27	16	76.19	22	95.65	6	85.71	
**CROWN-TO-IMPLANT RATIO**									
<2	26	50.98	5	23.81	14	60.87	7	100.00	0.01
2–2.99	16	31.37	7	33.33	9	39.13	0	0.00	
>2.99	9	17.65	9	42.86	0	0.00	0	0.00	

**Table 2:** Analysis of implant survival according to included study covariates. For all variables, values are presented as n (%).

VARIABLE	Implant Survival		Implant Failure		*p*-Value
	n	%	n	%	
**SEX**					
Male	20	100.00	0	0.00	0.51
Female	29	93.55	2	6.45	
**SMOKING**					
No	41	97.62	1	2.38	0.32
Yes	8	88.89	1	11.11	
**ASA STATUS**					
I	19	95.00	1	5.00	0.63
II	30	96.77	1	3.23	
**ORAL HYGIENE SESSIONS/YEAR**	3.47 ± 0.32		2.88 ± 0.65		0.15
**INTERPROXIMAL ORAL HYGIENE**					
No	10	100.00	0	0.00	0.64
Yes	39	95.12	2	4.88	
**HISTORY OF PERIODONTAL DISEASE**					
No	13	92.86	1	7.14	0.47
Yes	36	97.30	1	2.70	
**IMPLANT LENGTH**					
5 mm	21	100.00	0	0.00	0.62
6 mm	21	91.30	2	8.70	
8 mm	7	100.00	0	0.00	
**IMPLANT DIAMETER**					0.89
4 mm	2	100.00	0	0.00	
4.5 mm	14	93.33	1	6.67	
5 mm	28	96.55	1	3.45	
6 mm	5	100.00	0	0.00	
**TYPE OF TOOTH**					
Premolar	13	100.00	0	0.00	0.55
Molar	36	94.74	2	5.26	
**PROSTHETIC MATERIAL**					
Resin	6	85.71	1	14.29	0.25
Porcelain	43	97.73	1	2.27	
**CROWN-TO-IMPLANT RATIO**					
<2	25	96.15	1	3.85	0.74
2–2.99	15	93.75	1	6.25	
>2.99	9	100.00	0	0.00	

**Table 3:** RCBH (residual crestal bone height), IBHG (intra-sinus bone height gain), CBL (crestal bone level), and F-BIC (first bone-to-implant contact point). Values are presented as mean ± SD (max;min), or median(iqr) (max;min) at each time interval; SD = standard deviation; iqr = interquartile range.

	PRE-OPERATIVE	AFTER IMPLANT PLACEMENT	*p*-Value	AFTER LOADING	*p*-Value	AT 3-YEAR FOLLOW-UP	*p*-Value
**RCBH**	5.20(1.41) [10.66;2.74]	10.27(2.15) [15.08;7.81]	<0.001	8.88(2.35) [15.00;6.09]	<0.001	7.59(1.97) [14.27;5.23]	<0.001
**IBHG**		4.84 ± 1.38 [8.02;2.17]		3.96 ± 1.25 [6.33;1.19]	<0.001	3.17 ± 1.13 [6.01;0.76]	<0.001
**CBL**		1.87(0.93) [4.14;0.15]		1.46(0.81) [3.39;0.45]	<0.001	1.09(0.86) [3.24;−1.92]	<0.001
**F-BIC**				0.26(0.33) [1.08;−1.34]		0.37(0.45) [1.92;−0.31]	<0.001

**Table 4:** Comparison of RCBH, IBHG, CBL, and F-BIC at each time interval, between length groups. Values are presented as mean ± SD (max;min) or median(iqr) (max;min).

VARIABLE	Overall	5 mm	6 mm	8 mm	*p*-Value
**RCBH**					
Pre-operative	5.20(1.41) [10.66;2.74]	5.43(1.13) [10.66;2.97]	5.20(1.36) [10.30;2.74]	4.92(2.86) [7.68;4.07]	0.87
After implant placement	10.27(2.15) [15.08;7.81]	10.27(2.13) [15.01;8.01]	10.12(2.31) [15.08;7.95]	10.52(4.29) [13.53;7.81]	0.97
After loading	8.88(2.35) [15.00;6.09]	9.54(1.91) [12.16;6.34]	8.88(2.28) [15.00;6.09]	8.83(3.12) [10.84;6.26]	0.87
At 3-year follow-up	7.59(1.97) [14.27;5.23]	7.75(1.61) [11.92;5.23]	7.34(1.97) [14.27;5.69]	7.12(3.34) [9.58;5.94]	0.73
**IBHG**					
After implant placement	4.84 ± 1.38 [8.02;2.17]	4.74 ± 1.27 [8.02;3.01]	4.84 ± 1.48 [7.69;2.17]	5.12 ± 1.53 [7.5;3.35]	0.82
After loading	3.96 ± 1.25 [6.33;1.19]	3.81 ± 1.17 [6.2;1.98]	3.99 ± 1.36 [5.86;1.18]	4.3 ± 1.21 [6.33;3.01]	0.67
At 3-year follow-up	3.17 ± 1.13 [6.01;0.76]	2.95 ± 1.07 [6.01;1.59]	3.35 ± 1.27 [5.2;0.76]	3.22 ± 0.75 [4.69;2.2]	0.51
**CBL**					
After implant placement	1.87(0.93) [4.14;0.15]	1.89(1.22) [4.14;0.15]	1.84(0.78) [3.12;1.19]	1.87(1.01) [3.11;0.83]	0.98
After loading	1.46(0.81) [3.39;0.45]	1.70(0.80) [3.39;0.45]	1.38(0.81) [2.58;0.70]	1.23(0.61) [2.52;0.67]	0.28
At 3-year follow-up	1.09(0.86) [3.24;−1.92]	1.33(0.65) [3.24;−0.04]	1.09(0.86) [2.76;−1.92]	0.62(0.84) [2.43;−0.00]	0.2
**F-BIC**					
After loading	0.26(0.33) [1.08;−1.34]	0.26(0.40) [1.08;0.00]	0.25(0.33) [0.72;−1.34]	0.27(0.32) [1.04;−1.03]	0.9
At 3-year follow-up	0.37(0.45) [1.92;−0.31]	0.36(0.35) [1.42;0.00]	0.42(0.42) [1.92;−0.31]	0.37(0.43) [0.84;0.18]	0.59

**Table 5:** Comparison between satisfaction scores given seven days after surgery and given at three-year recall appointment. Unit of comparison was the patient. Values are presented as median(iqr) (max;min).

	PRE-OPERATIVE	AT 3-YEAR FOLLOW-UP	*p*-Value
**SATISFACTION SCORES**	8(2) [10;5]	9(1) [10;7]	<0.001

## Abbreviations

RCBH	Residual crestal bone height
OSFE	Osteotome sinus floor elevation
ISL	Internal sinus lift technique
ASA	American Society of Anesthesiologists
CBCT	Cone beam computed tomography
CIR	Crown-to-implant ratio
IAI	Implant-abutment interface
CBL	Crestal bone level
F-BIC	First bone-to-implant contact
∆-CBL	Average bone loss
∆F-BIC	Average apical shift of the first bone-to-implant contact point position
SFL	Sinus floor level
av	Average
IBHG	Intra-sinus bone height gain
BPPV	Benign paroxysmal positional vertigo
SD	Standard deviation
iqr	Interquartile range
